# The Nrf2 System as a Potential Target for the Development of Indirect Antioxidants

**DOI:** 10.3390/molecules15107266

**Published:** 2010-10-20

**Authors:** Kyeong-Ah Jung, Mi-Kyoung Kwak

**Affiliations:** College of Pharmacy, Yeungnam University, Gyeongsan, Gyeongsangbuk-do 712-749, Korea

**Keywords:** indirect antioxidants, oxidative stress, Nrf2, Keap1

## Abstract

Oxidative stress causes damage to multiple cellular components such as DNA, proteins, and lipids, and is implicated in various human diseases including cancer, neurodegeneration, inflammatory diseases, and aging. In response to oxidative attack, cells have developed an antioxidant defense system to maintain cellular redox homeostasis and to protect cells from damage. The thiol-containing small molecules (e.g. glutathione), reactive oxygen species-inactivating enzymes (e.g. glutathione peroxidase), and phase 2 detoxifying enzymes (e.g. NAD(P)H: quinine oxidoreductase 1 and glutathione-*S*-transferases) are members of this antioxidant system. NF-E2-related factor 2 (Nrf2) is a CNC-bZIP transcription factor which regulates the basal and inducible expression of a wide array of antioxidant genes. Following dissociation from the cytosolic protein Keap1, a scaffolding protein which binds Nrf2 and Cul3 ubiquitin ligase for proteasome degradation, Nrf2 rapidly accumulates in the nucleus and transactivates the antioxidant response element in the promoter region of many antioxidant genes. The critical role of Nrf2 has been demonstrated by various animal studies showing that mice with a targeted disruption of the *nrf2* gene are prone to develop lesions in response to environmental toxicants/carcinogens, drugs, and inflammatory insults. In this review, we discuss the role of the Nrf2 system, with particular focus on Nrf2-controlled target genes and the potential pleiotropic effects of Nrf2 activation of indirect antioxidants.

## 1. Introduction: Reactive Oxygen Species and Oxidative Stress

Reactive oxygen species (ROS) are constantly produced in aerobic organisms as by-products of normal oxygen metabolism and include free radicals such as superoxide anion (O_2_^−^) and hydroxyl radical (OH^-^), and non-radical hydrogen peroxide (H_2_O_2_). Superoxide anion is a common precursor of ROS and is involved in two pathways: i) rapid conversion into hydrogen peroxide and oxygen by superoxide dismutase (SOD) and ii) generation of highly toxic peroxynitrite via reaction with nitric oxide [1,2]. Further, hydrogen peroxide can be converted into hydroxyl radicals, particularly in the presence of transition metals such as iron and cobalt [3]. The mitochondrial respiratory chain and enzymatic reactions by NAD(P)H oxidase, xanthine oxidase, cyclooxygenases, and lipoxygenase, are endogenous sources of ROS [4]. Exogenous ROS-inducing stressors include ionizing radiation, UV light, and divergent oxidizing chemicals. 

At low concentrations, ROS serve as an important second messenger in cell signaling; however, at higher concentrations and long-term exposure, ROS can damage cellular macromolecules such as DNA, proteins, and lipids, which leads to necrotic and apoptotic cell death [4]. To restrict the potential toxicity of ROS, cells developed the antioxidant system. A nonenzymatic system involving thiol-containing small molecules such as glutathione (GSH) and thioredoxin (Txn) neutralizes ROS via direct interactions. An enzymatic system, including catalase, glutathione peroxidase (GPx), and peroxiredoxins (Prdx) reduce hydrogen peroxide to water. However, excess ROS can overwhelm the capacity of the antioxidant system, which leads to perturbation of cellular redox balance. Oxidative stress is a condition of imbalance between ROS formation and cellular antioxidant capacity due to enhanced ROS generation and/or dysfunction of the antioxidant system. Protein carbonyls, 8-hydroxyguanosine adducts, and lipid peroxides including 4-hydroxy-2-nonenal and isoprostane are biochemical markers of oxidative stress representing ROS-mediated damage to proteins, nucleic acids, and lipids [5]. Biochemical alterations in these macromolecular components can lead to various pathological conditions and human diseases: cancer, neurodegeneration, atherosclerosis, inflammation, and aging. In particular, ROS can stimulate cell proliferation, invasion, migration, and angiogenesis, and can evade apoptosis in cancer cells [6].

Recent discoveries in the cell biology of the cellular antioxidant system gave rise to the novel concept of “indirect antioxidants”. Indirect antioxidants act through the augmentation of cellular antioxidant capacity by enhancing gene expression. This review describes the role of the transcription factor Nrf2 in antioxidant gene regulation and its implications in various pathology and disease models. We suggest that small molecule Nrf2 activators may be a promising class of indirect antioxidants for the prevention/treatment of a wide array of human diseases. 

## 2. Cellular Antioxidant System

### 2.1. Directly Acting Antioxidant Proteins

Several proteins are directly involved in ROS removal. These include SOD, catalase, GPx, and small thiol molecules GSH and Txn. Among these, catalase, SOD, and GPx directly neutralize ROS. Mammalian catalase, with a molecular weight of 240 kDa is a tetramer of four identical subunits containing a porphyrin heme group [7] and is expressed in all tissues, but at particularly high concentrations in the liver, erythrocytes, and kidneys [8]. Catalase catalyzes the conversion of two molecules of hydrogen peroxide into two molecules of water and one molecule of oxygen in a decomposition reaction that can prevent the formation of highly reactive hydroxyl radicals from hydrogen peroxide. Human GPx is a selenoprotein which takes the form of 5 isotypes (GPx 1, 2, 3, 4, and 6), and can reduce hydrogen peroxide and soluble fatty acid hydroperoxides using two molecules of GSH as a co-substrate [9]. The antioxidative role of GPx has been demonstrated by gene knock-out studies in animal models: *GPx1* deficiency in mice led to abnormalities in endothelial and cardiomyocyte function due to severe oxidative stress [10].

GSH and Txn serve as substrates for GPx and Prdx. GSH is highly abundant (at millimolar concentrations) cellular tripeptide L-γ-glutamyl-L-cysteinyl-glycine and its biosynthesis occurs in the cytoplasm of most tissues via the action of γ-glutamate cysteine ligase (GCL) and GSH synthetase [11,12]. GCL is a heterodimer comprised of a catalytic subunit (GCLC) and a modulatory subunit (GCLM) [13]. Because of its high reactivity with free radicals, GSH is easily oxidized: biosynthesis and enzymatic reduction of disulfide by GSH reductase can rapidly supply GSH. Therefore, the ratio of GSH to oxidized GSH (GSSG) has been used as a marker of cellular redox status. Txn is located in the inner mitochondrial membrane and is involved in the reduction of hydrogen peroxide, lipid peroxide, and proteins with oxidatively modified sulfhydryl residues [14]. Txn reductase catalyzes the reduction of oxidized Txn using NAD(P)H, thereby maintaining a stable ratio of reduced to oxidized Txn. 

### 2.2. Phase 2 Detoxifying Enzymes as Antioxidant Proteins

Phase 2 detoxifying enzymes were originally recognized as xenobiotic metabolizing enzymes. Xenobiotics, which include various environmental chemicals, carcinogens, and drugs, undergo sequential two-step metabolism. Phase 1 enzymes primarily catalyze the introduction of functional groups into hydrophobic organic molecules through the action of cytochrome P450 enzymes [15]. Phase 2 enzymes are largely responsible for the elimination of xenobiotics by forming conjugated metabolites using hydrophilic molecules such as GSH and glucuronic acid. Phase 2 enzymes can be classified into four different categories: i) classical conjugating enzymes: glutathione *S*-transferases (GSTs) and UDP-glucuronosyl transferases (UGTs); ii) enzymes contributing to biosynthesis/recycling of thiols: GCL, GSH reductase, and Txn reductase; iii) enzymes involved in the reduction of reactive intermediates: NAD(P)H: quinine oxidoreductases (NQOs) and epoxide hydrolase (EH); iv) stress-response proteins: heme oxygenase-1 (HO-1) and ferritin [16,17]. Due to their role in maintaining redox balance, thiol homeostasis, and excretion of reactive metabolites (e.g., peroxides, epoxides, aldehydes, quinones), phase 2 detoxifying enzymes are now often classified as antioxidant proteins [16]. Further, because electrophiles can evoke GSH depletion and macromolecular damage, many environmental chemicals are regarded as oxidative stressors. One such example is a tobacco-related chemical benzo[*a*]pyrene (B[*a*]P): its reactive metabolic intermediate B[*a*]P-7,8 dihydrodiol-9,10 epoxide is a potent carcinogenic electrophile that depletes GSH and causes DNA damage [18].

GSTs are ubiquitous, multifunctional enzymes that detoxify endogenous and exogenous electrophiles, including epoxides, aldehydes, and peroxides. There are seven distinct classes of GSTs based on amino-acid sequence similarities, physical structure of the genes, and immunological cross-reactivity: alpha (α), mu (μ), omega (ω), pi (π), sigma (σ), theta (θ), and zeta (ζ) [19]. Animal knockout studies have revealed the functional role of GSTs in the susceptibility to environmental electrophiles. In *Gstp1/p2* null mice, the number of skin papillomas was significantly increased after exposure to the carcinogen 7,12-dimethylbenz(*a*)anthracene (DMBA) and 12-*O*-tetradodecanoyl-13-acetate (TPA) [20]. Lung cancer incidence after exposure to B[*a*]P was increased in *Gstp1-/-* mice [21]. In humans, cytosolic GSTs display polymorphisms which likely contribute to inter-individual differences in the response to xenobiotics and susceptibility to cancer [19]. The *Gstm1* null genotype is associated with an almost 2-fold higher risk for nasopharyngeal carcinoma in humans or animals [22].

UGTs catalyze the glucuronic acid conjugation reaction that mediates the major excretory pathway for pollutants and drugs, as well as endogenous compounds such as bilirubin, steroids, and hormones [23]. A number of studies have confirmed the protective role of UGTs against environmental chemicals and carcinogens: i) susceptibility to B[*a*]P carcinogenesis was enhanced in *ugt*-deficient cultured rat skin fibroblast [24]; ii) elevated UGT1A1 in breast cancer cells reduced DMBA-DNA adduct formation [25].

NQO1 (previous names include DT-diaphorase or quinone reductase type 1), a key enzyme belonging to the family of homodimeric flavoproteins, facilitates quinone excretion by catalyzing the reduction of quinones to hydroquinones through a single-step two-electron reduction reaction [26]. Since the alternative one-electron reduction of quinone can form semihydroquinone, which is capable of generating ROS through redox-cycling, NQO1 functions to prevent oxidative DNA damage by environmental stressors. Further, NQO1 plays an important role in preserving endogenous antioxidants by maintaining ubiquinone and α-tocopherol quinone in their reduced forms [27,28,29]. Therefore, mice with targeted disruption of *nqo1* were highly susceptible to B[*a*]P-induced mouse skin carcinogenesis [30]. NQO1*2, the C609T substituted polymorphic form of NQO1, has low NQO1 enzymatic activity compared with wild-type [31] and has been associated with greater risk of cancer incidence including pediatric leukemia [32], colorectal cancer [33], and gastric cardiac carcinoma [34].

HO-1 catalyzes the catabolic metabolism of heme to produce carbon monoxide and bilirubin [35,36]. Due to its role in removing the potent pro-oxidant heme and generating endogenous antioxidants carbon monoxide and bilirubin, HO-1 exhibits strong antioxidant capacity [36]. Ferritin, an iron storage protein, exhibits its antioxidative function by sequestering free iron that is potentially toxic when reacted with hydrogen peroxide [37,38]. 

## 3. Regulation of Antioxidant Genes by Nrf2

### 3.1. Antioxidant Response Element (ARE)

It has long been known that many phase 2 genes, including GSTs and NQO, are regulated through a *cis*-acting element, the antioxidant response element (ARE), located in their promoters. ARE was first identified in the 5′-flanking region of the rat *Gsta2* subunit gene (TAATGG*TGACAAAGC*A) [39] and this enhancer is essential for the inducible expression of *Gsta2* in response not only to phenolic antioxidants and metabolizable planar aromatic compounds, but also hydrogen peroxide and ROS [40]. Similarly, ARE (TCACAG*TGACTTGGC*A) of the rat *nqo1* gene is necessary for the inducible expression of this gene [41,42]; subsequently it was found that the rat *nqo1* ARE was highly conserved in the human *nqo1* gene (TCACAG*TGACTCAGC*A) [43]. In addition, *GCLC* and *GCLM*, which are genes encoding GSH biosynthetic enzyme subunits, contain multiple functional AREs; the human *GCLM* promoter retains tandem AREs, which are in opposite orientations [44,45]. In the promoter of the human *GCLC* gene, a reverse ARE (TCCCCG*TGACTCAGC*G) located at -3118 bp was identified as a functional ARE, responsible for induction in response to β-naphthoflavone and putative chemopreventive agents [46]. In an early study by Wasserman and Fahl *et al.* [47], the core sequence of ARE was proposed to be 5′-^A^/_G_TGA^C^/_G_NNNGCa/c-3′. However, as more AREs are identified in a wide array of phase 2 genes, great variability in the core sequence of ARE was found, indicating that consensus ARE sequences may be dependent on the specific interrogated gene. 

### 3.2. Nrf2 Signaling for the Regulation of ARE-Driven Genes

Based on the critical role of the ARE in antioxidant gene expression, the identification of transcription factors interacting with the ARE have long been a topic of interest. Extensive studies during the past decade have proven the notion that the transcription factor NF-E2-related factor 2 (Nrf2) is an essential element for regulation of the ARE [48,49,50,51]. Since the first demonstration that induction of *nqo1* and *gst* gene expression was abrogated in *t*-butylhydroxyanisole (BHA)-treated *nrf2*/- mouse [48], numerous studies have confirmed the crucial role of Nrf2 in the regulation of ARE-bearing genes in response to oxidants and electrophiles. Hepatic and gastric activities of GSTs and Nqo1 were reduced in *nrf2*-deficient mice compared with wild-type mice, and the induction of these enzymes by a chemopreventive agent, oltipraz, was almost blunt in *nrf2-/-* mouse [52]. When the dominant mutant Nrf2 was overexpressed, hemin-inducible expression of HO-1 was largely inhibited [53]. 

Many chemicals can induce the translocation of Nrf2 from the cytoplasm to the nucleus, where it can transactivate AREs with other bZIP transcription factor partners, including small Maf proteins (Maf F, Maf G, and Maf K) and ATF4 [51,54,55,56,57]. As Nrf2 activators, several phytochemicals, typical cancer preventive agents, GSH-depleting agents, electrophiles, and heavy metals are known to induce the expression of ARE-driven genes. In an attempt to elucidate common gene clusters regulated by Nrf2, several comparative analyses of global gene expression have been performed in *nrf2-/-* mice following treatment with enzyme-inducing chemicals. In the initial demonstration by Kwak *et al.*, the expression of 300 genes was increased by 3*H*-1,2,-dithiole-3-thione (D3T) treatment in wild-type mouse liver, whereas 77% of these inducible genes were not altered in *nrf2-/-* mice [58]. These Nrf2-dependent genes include clusters of phase 2 metabolizing enzymes such as GSTs and Nqo1, antioxidants, several cytochrome P450 enzymes, general enzymes, stress-response proteins, and the molecular chaperone-proteasome. Many of these Nrf2-dependent gene clusters were confirmed in other comparative gene analyses following treatment with isothiocyanates such as sulforaphane, phenolic antioxidants BHA and *t*-butyl hydroxyquinone (*t*BHQ), and synthetic oleanane triterpenoids [59,60,61,62,63,64,65,66], and representative antioxidant genes under the control of Nrf2 are summarized in Table 1. These comprehensive experimental approaches together with animal studies showing the loss of cytoprotective effect of enzyme inducers in *nrf2-/-* mice support the concept that Nrf2-target genes are primarily responsible for the advantageous action of phase 2 enzyme inducers. 

### 3.3. Keap1 as a Protein Inhibitor of Nrf2

**Figure 1:**
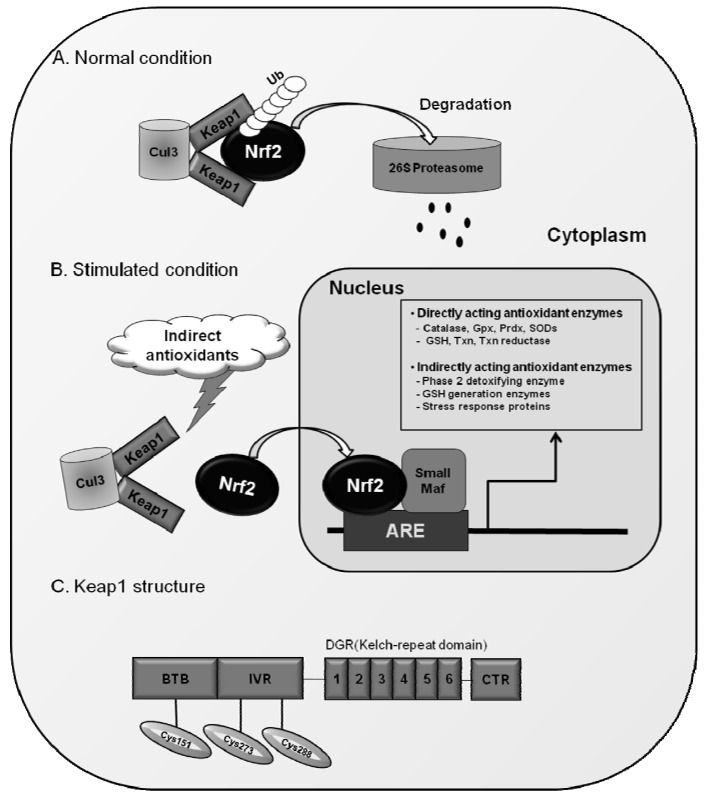
Nrf2 and its regulation by Keap1. (A) Under normal conditions, Nrf2 is degraded by Keap1-Cul3-dependent pathway; (B) In the presence of indirect antioxidants, Keap1-Nrf2 binding is disturbed and Nrf2 transactivates ARE-driven genes in the nucleus; (C) Protein structure of Keap1 and reactive cysteine residues for the action of Nrf2.

Nrf2 has six highly conserved homologous regions named Neh1 to Neh6 [80]. The Neh1 domain has a bZIP region interacting with partner proteins for heterodimerization [81]. The Neh3 domain interacts with chromodomain helicase DNA-binding protein 6 (CHD6) [82], and two acidic transactivation domains, Neh4 and Neh5, cooperatively bind with CBP (cAMP response element-binding protein-binding protein) [81]. The Neh2 domain located in the N-terminus of Nrf2 is known to be a regulatory domain responding to oxidative stress: Neh2 interacts with cytosolic protein, Kelch-like ECH-associated protein 1 (Keap1) and negatively controls Nrf2 function [83]. Keap1, which was originally isolated as an Nrf2-binding protein, is an actin-binding protein and has been thought to inhibit the function of Nrf2 by simply sequestering the protein in the cytoplasm [83,84]. However, as described in Figure 1, recent advances in Nrf2-Keap biology revealed that Keap1 functions as an adaptor protein between Nrf2 and Cul3, a component of the E3 ligase complex, and this binding promotes the continuous degradation of Nrf2 by the proteasome under normal conditions [85,86,87]. 

Since the dissociation of Nrf2 from Keap1 is the primary mechanism for Nrf2 activation, it will be an intriguing problem to determine how these two molecules interact with each other in different cellular environments. Recently, Yamamoto and colleagues have proposed the “hinge and latch” model to explain the response of Nrf2-Keap1 complex to stimuli [88,89]. Nrf2 contains 2 Keap1 binding sites within the Neh2 domain: DLG motif and ETGE motif, which enable the formation of a complex of one molecule of Nrf2 and two molecules of Keap1 [90,91]. Of note, these two sites have different binding affinities: the affinity of DLG motif to Keap1 is much weaker than that of ETGE. This led to the hypothetical “latch” binding of DLG-Keap1, which can be easily disturbed by Keap1 conformational changes of [88,89,90,91]. Since DLG binding involves the subsequent Cul3-proteasomal degradation of Nrf2, alterations in the DLG-Keap1 binding can result in the rescue of Nrf2 from degradation and accumulation of this protein within the nucleus. In recognition that Keap1 is a cysteine-rich protein (human and murine Keap1 contain 27 and 25 cysteines, respectively), modifications in sulfhydryl residues of Keap1 protein were initially speculated to result in protein conformational changes [83]. In fact, oxidative stress conditions and many exogenous chemicals alter the reducing status of cysteine residues of Keap1 and lead to Nrf2 translocation. As reactive cysteine residues mediating Nrf2 activation, an initial study by Dinkova-Kostova et al [92], has identified Cys257, Cys273, Cys288, and Cys297 to be dexamethasone-modified cysteine residues using mass spectrometry analysis. Subsequent independent studies confirmed that Cys273 and Cys288 are essential for Nrf2 activation in response to phase 2 enzyme inducers such as dithiolethiones and sulforaphane [91]. In addition to these, Cys151 was demonstrated to be required for the effect of sulforaphane and *t*BHQ [93,94,95]. Taken together, these results indicate that Keap1 is a sensor protein responding to oxidative and environmental stresses through dynamic changes in cysteine reducing status (Figure 1). 

## 4. Functional Role of the Nrf2 System: From Comparative Animal Studies

Since the first finding of Nrf2 as a master regulator of indirect antioxidant genes, various comparative animal studies using *nrf2-/-* mice have been performed to investigate the role of Nrf2 in the mammalian defense system. It is now widely accepted that cells and animals with a *nrf2* null genotype are much more sensitive to environmental or oxidative stress conditions, leading to accelerated macromolecular damage, mutations, and apoptosis. The initial study by Chan *et al*. [96], demonstrated that *nrf2-/-* mice are highly susceptible to butylated hydroxytoluene (BHT)-induced lung damage and lethality in comparison to wild-type mice. A following independent study showed that Nrf2 is a critical factor for determining susceptibility to hyperoxic concentration of oxygen-induced lung injury in mouse [97,98]. As a proof of its role in the CNS, astrocytes from *nrf2-/-* mice showed higher rates of cell death in response to hydrogen peroxide treatment [62]. Murine embryonic fibroblasts (MEFs) isolated from *nrf2-/-* mice showed higher levels of cell death in response to treatment with the redox-cycling ROS generator menadione and GSH-depleting anticancer agent cisplatin [99,100]. Incubation with diquat dibromide, another redox cycling bipyridylium herbicide, MEFs displayed markedly decreased cell viability, increased lipid peroxidation and GSH oxidation in comparison to wild-type cells [101].

Many electrophiles can cause oxidative stress leading to DNA mutations and carcinogenesis. As Nrf2 is a prime regulator for the expression of electrophile-detoxifying enzymes, the Nrf2 system has been recognized as a susceptibility determinant in response to chemical carcinogens. The first demonstration by Ramos-Gomez *et al.* showed that the incidence of gastric tumors was significantly increased in *nrf2-/-* mice following B[*a*]P treatment and B[*a*]P-DNA adduct levels were concomitantly increased in these mutant mice [52,102]. Aoki *et al.* demonstrated that exposure of *nrf2-/-* mice to diesel exhaust particles, which are postulated as a probable causal factor in lung cancer, resulted in higher levels of oxidative DNA damage with concomitant increases in lung injury [103]. The incidence of urinary bladder carcinoma by BBN was significantly higher in *nrf2-/-* mice than in wild-type mice [104]. When treated with arsenic, *nrf2-/-* mice showed more severe pathological changes in the liver and bladder, and arsenic-induced DNA hypomethylation was significantly elevated in the absence of *nrf2* [105]. 

Recent reports support the role of Nrf2 in the inhibition of inflammatory injuries. Following treatment with lipopolysaccharide (LPS), peritoneal neutrophils from *nrf2-/-* mice exhibited increased NADPH oxidase-dependent ROS generation and levels of TNF-alpha, IL-6 and chemokines (Mip2 and Mcp-1) compared to wild-type neutrophils [106]. *Nrf2-/-* macrophages were more susceptible to damage induced by reactive oxygen/nitrogen species, as well as acrolein and cadmium, macrophage toxins [107]. In addition to its role in inflammation, Nrf2 plays an inhibitory role in the fibrogenic process: bleomycin-induced pulmonary fibrosis and cyclosporine-mediated renal fibrosis were aggravated in *nrf2-/-* mice [108,109]. All together these reports confirmed the critical role of direct and indirect antioxidant proteins, which are under the control of Nrf2, for cytoprotection against divergent arrays of oxidative damage.

## 5. Indirect Antioxidants Activating the Nrf2 System

Given that Nrf2 primarily governs the expression of antioxidant genes, activation of Nrf2 signaling by specific chemicals can be conceded as one of effective means for prevention of oxidative injuries. In particular, naturally occurring chemicals from vegetables and fruits have been recognized to exhibit antioxidant, ROS-eliminating properties. β-Carotene is an example of direct antioxidants, while various phytochemicals exert their antioxidant activity through Nrf2 signaling. As recently reviewed by Surh *et al.* [110], phytochemicals activating Nrf2 signaling include isothiocyanates, dithiolethiones, resveratrol, curcumin, CAPE (caffeic acid phenethyl ester; from honeybee hives), epigallicatechin gallate (from green tea), allyl sulfides (from garlic), xanthohumols (from hop), and cinnamaldehyde. The beneficial effects of these phytochemicals have been demonstrated by numerous studies, and in particular, cancer preventive efficacy has been highlighted in many animal and human studies (Table 2).

The isothiocyanate sulforaphane (1-isothiocyanato-(4*R*)-(methylsulfinyl)butane) was isolated from broccoli by a bioassay screen of plant extracts for the identification of NQO1 inducers [128]. Accumulating lines of evidence show that sulforaphane is one of the most potent phase 2 enzyme inducers, and its indirect antioxidant properties have been demonstrated in various chemical-carcinogenesis animal studies. In female Sprague Dawley rats, oral administration of sulforaphane with doses of 75, 100, or 150 μmol/day for four days strongly inhibited tumor incidence, multiplicity, and burden by DMBA treatment [115]. In male Fisher rats treated with azoxymethane, sulforaphane inhibited the formation of colonic aberrant crypt foci [111]. Sulforaphane also inhibited lung adenomas induced by tobacco carcinogens [129] and suppressed the incidence of pancreatic tumors induced by *N*-nitroso-bis(2-oxopropyl)amine. Further studies with *nrf2-/-* mice have revealed that Nrf2 is the molecular target of sulforaphane. *Nrf2-*deficient mice lost antioxidant gene inducibility following sulforaphane treatment, and did not show the protective effect of sulforaphane in a B[*a*]P-induced gastric tumor formation model [113]. 

Dithiolethiones including D3T and oltipraz are organosulfur compounds [130]. Oltipraz, a congener of D3T, was initially developed for the treatment of schistosomiasis and has been extensively studied as a typical cancer chemopreventive agent. In various rodent organs, such as bladder, colon, kidney, liver, lung, pancreas, stomach, trachea, and blood, oltipraz pre-treatment inhibited carcinogenesis induced by divergent carcinogens including azoxymethane, aflatoxin B_1_, and B[*a*]P [116,117,118]. In addition, phase I and II clinical trials have demonstrated the cancer preventive efficacy of oltipraz in humans. A single dose of oltipraz (125~1,000 mg/m^2^) in humans increased enzymatic activity of NQO1, GCL, and GST in colonic mucosa [131]. NQO1 activity in peripheral mononuclear cells was also elevated in oltipraz-treated individuals without showing significant side effects. In a later randomized double-blind study in Qidong, People’s Republic of China, the intake of low-dose oltipraz increased the phase 2 conjugation reaction of aflatoxin B_1,_ indicating the facilitated detoxification of this potent carcinogen [132]. Anti-carcinogenic effects of dithiolethiones were found to be Nrf2-dependent: the induction of antioxidant genes by dithiolethiones (D3T and oltipraz) was blunt and cancer preventive efficacy of oltipraz has been lost in *nrf2*-deficient mice [50,52,58,104]. Keap1 has been proposed as the molecular target of dithiolethiones, and Wakabayashi *et al.* demonstrated that D3T led to intermolecular disulfide bond formation between Cys273 and Cys288 of two Keap1 proteins [91]. 

Resveratrol (3,5,4’-trihydroxystilbene) is a nonflavonoid polyphenol found in peanuts, grapes, and red wines [133]. Numerous studies have suggested that resveratrol in red wine can reduce the incidence of coronary heart disease and cancer [122]. Resveratrol has an intrinsic antioxidant property, which depends on the redox properties of phenolic hydroxyl groups [134], and its strong antioxidant properties were evidenced by the inhibition of polyunsaturated fatty acid oxidation, which is a core pathologic marker of atherosclerosis [135]. Recent reports showed that the protective effects of resveratrol are mediated by the activation of Nrf2: pretreatment with resveratrol protected H_2_O_2_-induced PC12 cell death and this protection was accompanied by upregulation of HO-1 [136]. Resveratrol restored cigarette smoke-depleted GSH levels by upregulating GCL expression through Nrf2 in human lung epithelial cells, and protected cells against cigarette smoke-mediated oxidative stress [137]. Further, enzymatic activities of antioxidant enzymes such as NQO1, Gpx, GR, GST, and SOD were increased after pretreatment with resveratrol in cultured hepatocytes [138].

Curcumin (1,7-bis(4-hydroxy-3-methoxyphenyl)-1,6-heptadiene-3,5-dione) is a major yellow pigment naturally isolated from the rhizomes of *Curcuma longa* and used as a coloring and flavoring agent [139]. Curcumin has various beneficial properties including anti-inflammatory, antioxidant, and chemopreventive activities [122,126]. Curcumin elevated enzymatic activity and protein levels of HO-1 through Nrf2 signaling in porcine renal epithelial proximal tubule cells (LLC-PK1) [140]. Dietary administration of curcumin in mice increased nuclear Nrf2, ARE binding activity, and target gene expression in the liver and lungs. As a consequence, curcumin-treated mice showed significantly reduced DNA adduct formation, oxidative damage, and inflammation following B[*a*]P challenge [141]. 

## 6. Pleiotropic Effects of Small Molecule Nrf2 Activators

Health benefits of statins in reducing cardiovascular risk are not solely dependent on their cholesterol-lowering effects: basic and clinical studies have demonstrated that the inhibition of inflammation, maintenance of endothelial function, and modulation of platelet function cooperatively contribute to the protective effect of statins [142,143]. Based on this concept, it has been widely speculated that the use of statins can prevent/ameliorate stroke, Alzheimer’s and renal diseases, and cancers [143,144,145]. Recent advances in understanding the mechanism of action of statins suggest that these non-cholesterol beneficial effects can be explained by a single mechanism inhibiting 3-hydroxy-3-methylglutaryl coenzyme A (HMG CoA reductase) and the consequent reduction in isoprenoids, which are essential for the posttranslational modification of several signaling proteins. Small molecule Nrf2 activators already gained attention for their cancer preventive efficacy. However, recent understanding of the gene clusters regulated by Nrf2 and its various pathophysiological implications provided in-depth characterization of Nrf2 signaling, indicating the pleiotropic effects of Nrf2 activators.

### 6.1. Anti-Inflammatory Function of Small Molecule Nrf2 Activators

Considerable evidence supports the notion that Nrf2 activation can suppress the inflammatory response although its mechanism has not been fully defined. As increased ROS generation is a core event of inflammation, enhanced cellular antioxidant capacity is believed to contribute to the repression of inflammatory injuries. In particular, potent anti-inflammatory function of HO-1 has been highlighted: increased carbon monoxide, an inhibitor of macrophage activity, is thought to participate in the anti-inflammatory role of HO-1 [35,146,147]. Consistent with these reports, a number of studies have shown that indirect antioxidant-mediated Nrf2 activation is strongly associated with the protection of animals from pro-inflammatory insults [148]. Indeed, a potent Nrf2 activator synthetic triterpenoid significantly reduced LPS-induced cytokine production and in turn suppressed mortality by LPS in wild-type mice; however these protective effects were not observed in *nrf2*-null mice [106]. In the context of chronic inflammation and its causal link to carcinogenesis, the anti-inflammatory role of small molecule Nrf2 activators appears to be one of mechanisms of cancer-preventive action. 

### 6.2. Modulation of Proteasome Function by Nrf2 Activators: Implication in Protein Toxicity-Associated Diseases

A comparative global gene expression analysis in *nrf2-/-* mice led to the identification of multiple subunits of hepatic 26S proteasome as D3T-inducible, Nrf2-dependent genes [58]. In a subsequent study, it was shown that the expression of Psmb5, which is the catalytic core subunit of the 20S proteasome, can be up-regulated by proximal AREs [149]. In addition to increased expression of individual subunits, total enzymatic activity of the proteasome was significantly enhanced in multiple tissues from D3T-treated mice including liver, lung, kidney, and small intestines [150]. Since the proteasome, the 20S proteasome in particular, is known to be largely responsible for the degradation of oxidatively damaged proteins, under conditions with abnormal proteasome function, cells will be encountered by condition termed protein toxicity [151,152,153]. This protein toxicity has been highlighted in neurodegenerative diseases such as Alzheimer’s disease and amyotrophic lateral sclerosis. Therefore, the use of Nrf2 activators may prevent protein toxicity by maintaining proteasome function. In fact, sulforaphane treatment ameliorated hydrogen peroxide-induced protein oxidation and resulted cytotoxicity by modulating proteasome expression in murine neuroblastoma cells [154]. Degradation of SOD1 G93A protein, which has been postulated as an ALS-causing mutant protein, was facilitated in tissue homogenates from D3T-treated mice [150]. 

### 6.3. Lipid Metabolism and Nrf2 Activators.

Several recent findings indicated a potential role for Nrf2 in lipid metabolism. For instance, Shin *et al.*, reported that *nrf2*-null MEFs showed markedly accelerated adipogenesis, which was triggered by rosiglitazone treatment, compared to wild-type MEFs [155]. In these cells, treatment with an Nrf2-activating triterpenoid effectively inhibited adipocyte differentiation; however *nrf2*-null cells were unaffected by triterpenoid. In a follow-up study, another synthetic triterpenoid 1-[2-cyano-3-,12-dioxo-oleana-1,9(11)-dien-28-oyl]imidazole (CDDO-Im) completely prevented the increase in weight gain of wild-type mice imparted by a high-fat diet, whereas *nrf2*-disrupted mice were not responsive to the preventive intervention of CDDO-Im [156]. Similar findings have been reported by Tanaka *et al.*, and concentrations of free fatty acid and malondialdehyde equivalents were higher in *nrf2*-null mice compared with wild-type mice after four weeks on a high-fat diet [157]. 

### 6.4. Liver Regeneration and Nrf2 

Another promising effect of Nrf2 activators is their potential role in liver regeneration after injury. A recent study by Beyer *et al.* demonstrated that liver regeneration after a partial hepatectomy is significantly delayed in *nrf2*-null mice compared to wild-type mice [158]. This finding has been explained by the observation that increased oxidative stress in the *nrf2*-null condition can lead to insulin/insulin-like growth factor resistance and further impairment of p38 MAPK and AKT signaling, which is necessary for liver cell proliferation [159]. A similar finding was reported by Wakabayashi *et al.*; however, this group highlighted the direct role of Nrf2 in the regulation of Notch1, suggesting that a loss of *nrf2* impedes Notch function and thereby interferes with liver cell proliferation [160]. These results suggest that Nrf2 activators may have beneficial effects in recovery from liver damage, which can result from chronic alcohol consumption, chemical toxins, and drug/viral inflammation. In fact, this possibility has been supported by Kang *et al.*: oltipraz treatment of rats with established cirrhotic liver enhanced regeneration and reduced cirrhotic nodules with a concomitant improvement in survival [161,162]. Although the authors concluded that activation of CCAAT/enhancer binding protein β (C/EBPβ) and a consequent suppression of TGFβ expression is a primary mechanism of oltipraz-mediated regeneration, the potential involvement of Nrf2 signaling may be of interest for future investigations. 

## 7. Conclusions

The utility of direct antioxidants has been confronted by several randomized clinical studies showing that vitamins C, E, and β-carotene do not reduce cancer incidence in humans. In addition, due to their short half-lives, direct antioxidants need to be administered frequently and relatively high dosages are required to sustain their physiological efficacy. Indirect antioxidants are defined as small molecules that can upregulate the expression of genes encoding antioxidant proteins through Nrf2. Eventually, this effect influences the physiological, biochemical, and/or cellular processes that inactivate free radicals or that prevent free radical-initiated chemical reactions. In contrast to the short half-life of direct antioxidants, indirect antioxidants act through gene regulation, so their physiological effects last longer than do those of direct antioxidants. Furthermore, indirect antioxidants are unlikely to evoke pro-oxidant effects, which have been a problem in the use of high dose vitamin E therapy. Of note, accumulating lines of evidence suggest that small molecule Nrf2 activators exert pleiotropic effects: prevention of cancer, amelioration of inflammatory injuries, protection against protein toxicity, promotion of liver regeneration after injuries, and maintenance of balanced lipid metabolism. 

As for the development of indirect antioxidants, Keap1 is now accepted as a direct molecular target of antioxidant gene inducers. However, at this time, due to the limited understanding of the structural biology of Keap-Nrf2 proteins, the detailed mechanism of how specific chemicals react with specific sulfhydryl residues of the Keap1 protein remains to be uncovered. In general, inducers of antioxidant genes are diverse in their structures and chemical properties; however, one common feature is the high reactivity to sulfhydryl groups through the oxidation or alkylation reaction. Originally, Paul Talalay and colleagues defined 9 different chemical classes of sulfhydryl-reactive gene inducers, including isothiocyanates, dithiolethiones, a variety of Michael reaction acceptors, arsenicals and heavy metals, hydroperoxides, vicinal dimercaptans, oxidized diphenols, phenylene diamines, and quinones [163]. A recent study by Kobayashi *et al.*, developed a zebrafish model of a Keap1 mutation, and classified several sulfhydryl reactive chemicals into distinct categories depending on the reactive cysteine residues required for their action [164]. In their classification, sulforaphane, D3T, and GSH depletor diethylmaleate are classified into the same class based on the requirement of Cys151 for Nrf2 activation, while Prostaglandin A_2_ and 15-deoxy-Δ^12,14^-prostaglandin J_2_, in a different class, require Cys273 for their action. On the other hand, an independent study reported that hydrogen peroxide modified multiple cysteine residues of Keap1, including Cys77, Cys297, Cys319, Cys369, and Cys434, indicating more nonspecific modifications in this case [165]. These suggest a clear notion that particular cysteine residues of the Keap1 protein respond to differential signals in a specific way. Therefore, an accurate understanding of the cysteine reactivity of the Keap1 protein will promote the development of more specific antioxidants for the activation of Nrf2. In conclusion, we propose that the development of specific small molecule Nrf2 activators might be a successful strategy to control or prevent a wide array human disease, which is associated with oxidative injuries. 

## Figures and Tables

**Table 1:** Antioxidant genes regulated by Nrf2.

Function	Gene	Species (organs)	Reference

GSH biosynthesis	GCLC	Mouse (liver, lung) Human (HaCaT;keratinocyte)	[58,67] [68]
	GCLM	Mouse (liver, lung) Human (IMR-32;neuroblastoma cell) (HaCaT)	[58,67] [68,69]
	GSR	Mouse (liver, lung) Human (IMR-32, HaCaT)	[70,71] [68,69]
Glutathione peroxidases	GPx1	Mouse (cardiovascular, lung)	[72]
	GPx2	Mouse (liver, lung) Rat (liver) Human (Caco-2;colon Cell) Rat (liver)	[73,74] [75] [76]
Thioredoxin reductase	TXNRD	Mouse (liver, lung) Human (IMR-32)	[65,67] [69]
Thioredoxin	TXN	Mouse (liver)	[58]
Peroxiredoxin	PRDX1	Mouse (liver,lung)	[67,74]
	PRDX6	Human (A549; lung derived cell line)	[77]
Superoxide dismutase	SOD1	Mouse (liver)	[71]
	SOD2	Mouse (liver)	[71]
	SOD3	Mouse (lung)	[67]
	Catalase	Mouse (liver, lung)	[71,73]
Glutathione S-transferases	GSTA1	Mouse (liver, lung, small intestine)	[64,67,71]
	GSTA2	Mouse (liver, lung, small intestine)	[58,64,67]
	GSTA3	Mouse (liver, lung, small intestine)	[64,67,71]
	GSTA4	Mouse (liver)	[65]
	GSTM1	Mouse (liver, small intestine)	[58,64,65]
	GSTM2	Mouse (liver, small intestine)	[58,64,65]
	GSTM3	Mouse (liver, small intestine) Human (IMR-32)	[58,64,65] [69]
	GSTM4	Mouse (liver)	[65]
	GSTM5	Mouse (liver)	[59]
	GSTM6	Mouse (liver)	[65]
	MGST2	Mouse (small intestine)	[64]
	MGST3	Mouse (liver, small intestine)	[58,64]
UDP-glucuronosyl transferase	UGT1A6	Mouse (liver)	[74]
	UGT2B1	Mouse (liver)	[71]
	UGT2B5	Mouse (liver, small intestine)	[58,64]
Reduction	NQO1 AKR1A AKR1B8	Mouse (liver, lung, small intestine) Human (IMR-32) Mouse (liver, small intestine) Mouse (liver, small intestine)	[58,64,67] [69] [58,64] [64,67]
Heme oxygenase-1	HO-1	Mouse (liver) Rat (liver) Human (IMR-32, HaCaT)	[59,76] [68,69]
Hydrolysis	EPHX	Mouse (liver, small intestine)	[58,64]
Iron transport	Ferritin H	Mouse (lung) Human (HaCaT)	[73] [68]
	Ferritin L	Mouse (liver) Human (HaCaT)	[65] [68]
Detoxication of heavy metals Transport	MT І	Mouse (embryonic fibroblasts) Human (HepG2 cell;hepatoma)	[56] [78]
	MT ІІ	Mouse (embryonic fibroblasts) Human (HepG2 Cell)	[56] [78]
	MRP2	Mouse (liver)	[71]
	MRP3	Mouse (liver) Human (NSCLC, HBE1)	[71] [79]
26S Proteasome	Psma1,4,5,6,7 Psmb1,2,3,4,5,6	Mouse (liver) Mouse (liver)	[58] [58]
	Psmc1,3 Psmd1,5,7,11,12,13	Mouse (liver) Mouse (liver)	[58] [58]

**Table 2:** Indirect antioxidants and their effects on chemical toxicity.

Indirect antioxidants	Effect on target organ toxicity
Sulforaphane 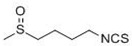	Protection against tumor formation induced by many carcinogens: mammary, colon, lung, pancreatic, gastric, intestine, skin, and bladder (mouse, rat, hamster) [16,111,112,113,114,115]
D3T 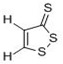	Inhibition of aflatoxin B1 induced hepatic tumorigenesis (rat) [116,117].
Oltipraz 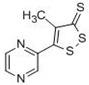	Inhibition of carcinogenesis induced by various carcinogens in bladder, colon, kidney, liver, lung, pancreas, and stomach (mouse, rat) [116,118,119].
Resveratrol 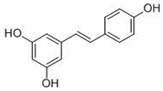	Inhibition of growth of variety tumors: skin, breast, gastric, colon, small intestine, lung, esophageal, prostate, liver, and pancreatic cancers (mice, rat) [120,121,122]. Human clinical trials in breast cancer patients [123].
Curcumin 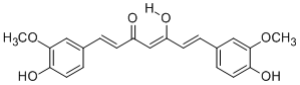	Inhibition of tumor development in skin, liver, oral, esophageal, stomach, intestinal, colon, and bladder (mouse, rat) [124]. Human clinical trials in patients with advanced pancreatic cancer and other disease [125,126].
CAPE 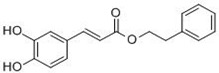	Anti-proliferation property in cancer cells [127].
